# Review of Ultrasonic Ranging Methods and Their Current Challenges

**DOI:** 10.3390/mi13040520

**Published:** 2022-03-26

**Authors:** Zurong Qiu, Yaohuan Lu, Zhen Qiu

**Affiliations:** 1 State Key Laboratory of Precision Measuring Technology and Instruments, Tianjin University, Tianjin 300072, China; luyaohuan@tju.edu.cn; 2 School of Engineering, Faculty of Engineering and Technology, Liverpool John Moores University, Liverpool L3 3AF, UK; z.qiu@ljmu.ac.uk

**Keywords:** ultrasonic ranging, transducer, pulse echo, time of flight, error compensation

## Abstract

Ultrasonic ranging has been widely used in automobiles, unmanned aerial vehicles (UAVs), robots and other fields. With the appearance of micromachined ultrasonic transducers (MUTs), the application of ultrasonic ranging technology presents a more extensive trend. This review focuses on ultrasonic ranging technology and its development history and future trend. Going through the state-of-the-art ultrasonic ranging methods, this paper covers the principles of each method, the signal processing methodologies, the overall system performance as well as key ultrasonic transducer parameters. Moreover, the error sources and compensation methods of ultrasonic ranging systems are discussed. This review aims to give an overview of the ultrasonic ranging technology including its current development and challenges.

## 1. Introduction

An ultrasonic transducer is a device that can realize the mutual conversion between high-frequency electrical energy and mechanical energy. It is generally divided into the piezoelectric type, capacitive type and magnetoelastic type and is widely used in measurement fields, including distance measurement (in the air) [1], sonar [2], medical imaging [3,4], ultrasonic therapy [5], non-destructive testing [6,7], flow monitoring [8,9], etc. Ultrasonic ranging has the advantages of relatively low hardware requirements over radio frequency ranging and laser ranging in short-range measurement due to ultrasound’s slower transmission speed. Besides, ultrasonic is not sensitive to ambient light, electromagnetic interference, dust or other factors. In addition, the relatively low cost of ultrasonic transducers is user-friendly for engineering applications such as reversing radar and construction surveys. Therefore, ultrasonic ranging is suitable for small range (the distance is usually less than 10 m) and high-precision (the accuracy can reach the level of millimeter generally) non-contact distance measurement, and the derived two-dimensional positioning [10] and three-dimensional positioning [11,12], object shape recognition [13] and multi-sensor fusion trajectory measurement [14], etc.

Ultrasonic ranging originated with underwater sonar in the 1940s, and as it was developed for use in the air in the 1960s, possibilities for contactless distance measurement from 0.2 m to 2 m in the air opened up [15]. In the 1980s, the research and application of ultrasonic ranging systems gradually began and was first applied to robot obstacle avoidance [16,17,18,19]. Since the 1990s, the research on ultrasonic ranging methods has gradually deepened, and not only does the traditional time of flight (ToF) method continues to be studied [20,21], but two frequency continuous wave (TFCW) method [22], multi-frequency continuous waves (MFCW) method [23], binary frequency shift keying (BFSK) method [24] and amplitude modulation (AM) method [25] have also been proposed, different new measurement methods continue to emerge. Since the beginning of this century, MEMS ultrasonic devices have been gradually applied to ultrasonic ranging, and different signal processing methods have been gradually developed including thresholding method, curve-fitting method, sliding-window method and optimum correlation detection in [26]. Different coding methods have also been gradually applied, developing ultrasonic ranging systems towards miniaturization, high precision and high processing speed. At present, in addition to improving the performance of ultrasonic devices and ultrasonic systems, there is also optimization of the processing algorithm aimed at the high performance and wide applications of ultrasonic ranging systems.

Nowadays, ultrasonic ranging is widely used in the automotive industry, UAVs, robots, and industrial auxiliary measurement equipment. In the automotive industry, it is mainly used for the detection of obstacles to assist the driving system with achieving anti-collision function, including distance measurement to obstacles [27,28] and acquisition of kinematics information for obstacles moving around the vehicle [29]. Additionally, the type of road surface can be identified through reflected signals [30] to assist in safe driving. Similarly, ultrasonic sensors can also be applied to obstacle avoidance of UAVs [31,32] and robots [33]. In addition, it can also be used to detect the landing state [34] to ensure the flight safety of UAVs. In the field of robots, indoor positioning is also a common application. Whether position coordinate measurements [35] or attitude measurement [36], it is realized through the fusion of multiple transmitters in the space and multiple receivers on the robot, and the positioning accuracy can reach the level of centimeters [37]. Meanwhile, the auxiliary navigation system can realize the path planning of the robot [38]. In the industrial field, ultrasonic equipment can be used to monitor the field environment such as the tower crane in [39] to ensure the normal operation of industrial equipment.

Conventional bulk piezoelectric ultrasonic transducers are not practical in mobile applications due to their large size and high power consumption compared to micromachined ultrasonic transducers (MUTs) [40]. MUTs can be integrated into portable products, such as smartphones, wearable devices, etc. As a result of research and technical development, the performance of MUTs has been improved, and they are hoped to replace the buck piezo-composite ultrasonic transducers. For example, multiple ultrasonic sensors can be integrated into mobile devices for distance estimation, which could then be combined with other information of reflected ultrasonic signals to realize gesture recognition and classification [41,42], to achieve contactless human–computer interaction. In addition, a sliding-window based method [43] can be used to improve accuracy. Therefore, MUTs can be applied to smartphones, VR devices and smart home products.

In this study, the status of ultrasonic ranging is reviewed from different aspects. Section 2 introduces the characteristic parameters of ultrasonic transducer related to ranging application, including frequency and impedance, energy conversion characteristics, and ultrasonic propagation characteristics, and also analyzes the influence on ranging systems. Section 3 presents the principle, composition and evaluation parameters of ultrasonic ranging systems. Section 4 summarizes the various measurement methods and signal processing methods, as well as the corresponding measurement range, measurement accuracy and measurement rate. Section 5 focuses on the error source and error compensation method of ultrasonic ranging. In the final section, conclusions are drawn, existing problems are summarized, and predictions of the future development trend are provided.

## 2. Transducer and Ultrasonic Characteristics Related to Ranging

### 2.1. Comparison of Different Transducers 

**Figure 1:**
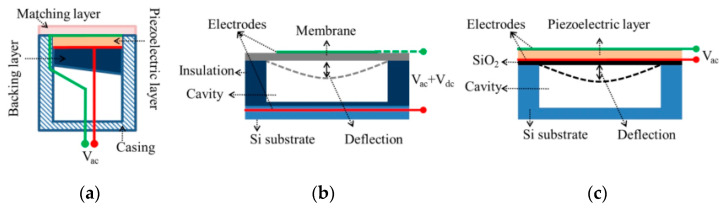
Typical cross-sectional structures of (**a**) piezoelectric ultrasonic transducers; (**b**) CMUTs and (**c**) d31-mode PMUTs [51].

An ultrasonic transducer is a kind of energy conversion device, which converts mechanical energy to electrical energy or vice versa. Although conventional bulk piezoelectric transducers can generate high output power, acoustic impedance mismatchs between the devices and transmission medium severely reduce transduction efficiency [44]. A typical structure of a conventional piezoelectric ultrasonic transducer is shown in Figure 1a. In order to reduce the mismatch of acoustic impedance between the piezoelectric layer and the propagation medium, a matching layer usually is added at the transducer’s front face [45,46,47,48,49]. For the transducers operating with an air load, the best properties of a matching layer are observed from the materials of polyether sulfone and nylon membranes [47]. A matching layer based on a combination of a porous material with a low-density rubber material can achieve an improvement in received signal amplitude of 30 dB when compared with the unmatched case [48]. In addition, transducers based on ferroelectrets have been reported with reduced impedance mismatch with the air [50].

In contrast to conventional ultrasonic transducers, the MUTs employ a flexural membrane for generating and receiving ultrasound waves, which is conducive to better impedance matching and higher transmitting efficiency [51]. Therefore, MUTs are mainly divided into capacitive micromachined ultrasonic transducers (CMUTs) [52] and piezoelectric micromachined ultrasound transducers (PMUTs) [53]. CMUTs’ operation is based on the flexural vibrations caused by a field-induced electrostatic attraction between the suspended membrane and the substrate (Figure 1b), whilst PMUTs is based on flexural vibrations caused by d31- or d33-mode excitation of a piezoelectric membrane (Figure 1c).

CMUTs have the advantage of high bandwidth (often over 100%) [51], which is more conducive to the modulation of the ranging system. However, the output pressure of CMUTs depends on the excitation voltage and the inverse of the capacitor gap [54]. Hence, the CMUTs usually have submicrometer gaps [55] which leads to not only a complicated fabrication process but also a small linear vibration amplitude. To overcome this problem, high bias voltages of hundreds of volts can be used [56], which, however, would lead to further increase in the system complexity and higher power consumption. 

Compared with CMUTs, PMUTs do not require DC bias voltage [57,58], and the linear displacement range is a function of the membrane thickness [59]. For PMUTs, the active piezoelectric layer, e.g., lead zirconate titanate (PZT) and aluminum nitride (AlN), is deposited with nanofabrication techniques, e.g., plasma-enhanced chemical vapor deposition (PECVD), sol-gel process, and sputtering, on passive elastic layers such as Si, Si_x_N_y_ or SiO_2_. The resonant frequency of the PMUTs does not directly depend on the thickness of the piezoelectric layer. Instead, the flexural mode resonant frequencies are closely related to the shape, dimensions, boundary conditions, intrinsic stress and mechanical stiffness of membranes [53]. In practical applications, PMUTs can generate sufficient ultrasonic power from a sub-mW electrical drive signal for target ranges up to a few meters [60] and can meet the requirements of output pressure in the range. Therefore, PMUTs are expected to be a better solution for rangefinders [61] due to their superior power efficiency even though that is less than conventional bulk transducers. As an example, a power dissipation of 400 μW at 30 fps for a 1 m maximum range has been achieved with an AlN-based ultrasonic array transducer [62]. The comparisons of bulk piezoelectric transducers, CMUTs and PMUTs are shown in Table 1.

### 2.2. Transducer Characteristics 

The following subsections describe some characteristics of transducers related to the ultrasonic ranging system’s performance.

#### 2.2.1. Frequency Characteristics

The operating frequency of the ultrasonic ranging transducer is often chosen to be near the series resonant frequency which has minimal impedance. The frequency bandwidth of PMUTs used for ranging in the air is generally narrow, e.g., 25% in [63], compared with CMUTs, >100% reported in [61]. Current ranging systems generally use a certain frequency of the transducer rather than frequency modulation. The double resonant frequency transducer [64] is also under study, which can obtain a broadened frequency response. Broader bandwidth allows the adoption of the frequency modulation operation and is conducive to the improvement of ranging technology. In addition, the operating frequency determines ranging accuracy. In general, the higher the working frequency, the higher the ranging accuracy. However, this will be at the expense of the ranging range as the attenuation of ultrasound in the propagation medium will increase proportionally to the frequency [65]. Therefore, the choice of operating frequency should be matched with specific measurement requirements.

#### 2.2.2. Impedance

**Figure 2:**
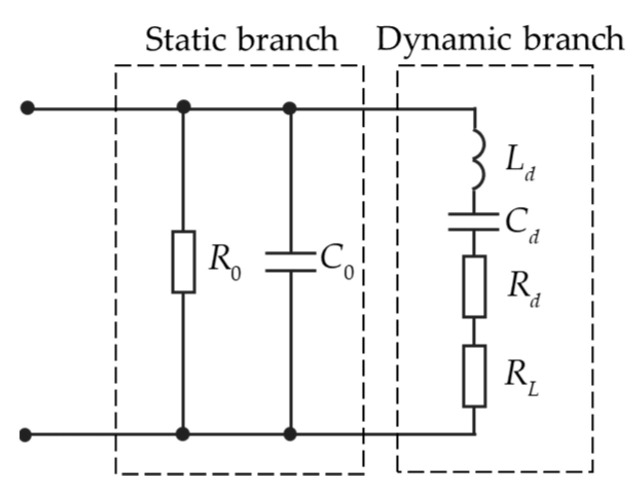
Equivalent circuit model of transducer.

Impedance analysis of transducers can evaluate the dynamic characteristics of transducers [66]. At present, most ultrasonic transducers are made of polycrystalline piezoelectric ceramic materials. High-frequency electrical signals, >20 kHz, are applied to piezoelectric materials and converted into ultrasonic signals. The equivalent circuit model of the piezoelectric ultrasonic transducer is shown in Figure 2.

Where $R_{0}$ is the parallel resistance of medium loss, $C_{0}$ is the static capacitance, measured far below the resonant frequency, $C_{d}$ is dynamic capacitance, $L_{d}$ is dynamic inductance, $R_{L}$ is the load resistance. In general, $R_{0}\gg R_{d}$. In order to simplify the model, $R_{0}$ can be ignored during analysis. In addition, $R_{L}$ is short-circuited during analysis. Then, the equivalent impedance of the piezoelectric transducer can be expressed as Equation (1) [67]

(1) $$Z=\frac{(R_{d}+j\omega L_{d}+1/j\omega C_{d})\cdot 1/j\omega C_{0}}{R_{d}+j\omega L_{d}+1/j\omega C_{d}+1/j\omega C_{0}}$$

To maximize the power transmission efficiency of the ranging system, make the dynamic branch in resonance, and the resonant frequency $\omega =\omega_{s}=1/\sqrt{L_{d}C_{d}}$. Then the impedance of the transducer can be shown as Equation (2), as a combination of both resistance and reactance.

(2) $$Z=R+jX=\frac{R_{d}}{1+{\left(\omega_{s}C_{0}R_{d}\right)}^{2}}-j\frac{\omega_{s}C_{0}R_{d}^{2}}{1+{\left(\omega_{s}C_{0}R_{d}\right)}^{2}}$$

Matching circuits are commonly included in the ultrasonic system, aiming to make the transducer closer to pure resistance in order to achieve higher power output and better efficiency [68,69]. An improvement of 300% in the amplitude of the received signal was reported with and without the matching circuit [68]. This improvement leads to an improved signal to noise ratio (SNR) and, therefore, better accuracy in measurements which is desirable in the ultrasonic ranging system.

#### 2.2.3. Electromechanical Coupling Coefficient

The electromechanical coupling coefficient $k^{2}{}_{eff}$ is a parameter of ultrasound transducers to reflect energy conversion efficiency. A higher $k^{2}{}_{eff}$ is desired to have more energy to be converted between electrical and mechanical energy within the transducer [4]. The $k^{2}{}_{eff}$ can be calculated using Equation (3) [70]

(3) $$k^{2}{}_{eff}=1-{\left(\frac{f_{r}}{f_{a}}\right)}^{2}$$

where $f_{r}$ is the resonance frequency and $f_{a}$ is the anti-resonance frequency.

In general, the $k^{2}{}_{eff}$ of PMUTs used is less than 5%, and the method called Clarinet^TM^ to increase the coefficient is also being studied [71]. Due to the high attenuation in ultrasound waves along the travelled path in the air, the ultrasonic ranging system would benefit from a transducer with a higher coupling coefficient to improve its signal sensitivity and measurement accuracy.

Apart from optimizing for a higher coupling coefficient of the transducer, other approaches are used to improve the SNR of the received signals as well for the ranging system. In [72], the authors reported that the signal amplitude of the bent transducer was enhanced 10 times bigger by designing its active diameter. The signal quality can also be improved by optimizing the excitation voltage of the transmitting transducer [73], exciting the transmitter transducer with a direct modulation system [74] and adopting the broadband tuning of the receiving circuit [75]. 

#### 2.2.4. Directivity

The directivity of transducer and transducer array is a characteristic that the amplitude of transmitting response or receiving response changes with azimuth angle. It determines the range of the azimuth angle of the measurable range in the space. Depending on their applications, the requirements for directional characteristics of transducers are different. For a transmitting transducer, the sharpness of its directional characteristic curve determines the concentration of its transmitted energy, and for a receiving transducer, it determines the range of azimuth to explore space. In the ultrasonic ranging system, the transducers can be used as either the transmitter, the receiver or both depending on the configurations. 

The sound field established by the transducer in the medium is related to the shape and size of the transducer, the vibration mode, the working parameters such as frequency and the types of the medium [76]. 

**Figure 3:**
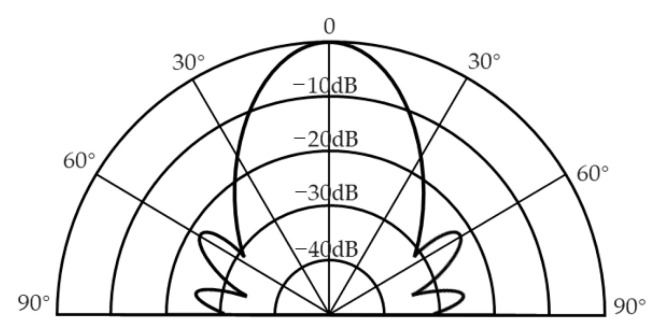
Typical directivity diagram of a transmitting ultrasonic transducer.

The directivity response diagram can be obtained by drawing the directivity response diagram in decibel (dB) compared to its maximum in the sound field. Figure 3 presents the directivity diagram of a transmitting ultrasonic transducer. 

As shown in Figure 3, there are the main lobe and sidelobes in the acoustic field [77], with the main lobe mainly could be used for ranging. In applications where the ranging direction is fixed, the transducer is generally configured in an orientation to align with its main lobe’s direction. 

When the width of the main lobe becomes narrower, the energy of the beam is more concentrated and its directivity is higher. The parameter, beam spread angle θ, can be used to represent the directivity of the ultrasound transducer. Beam spread angle measures the width of the beam in degrees, from side to side of the main lobe where the ultrasonic energy intensity drops to −3 dB. In general, the ranging system takes the corresponding space within the beam spread angle of the transducer as the detectable range.

When the beam spread angle of a single-element ultrasonic transducer cannot meet the range requirement, transducer array and beam forming techniques can be adapted to change the directivity of transducers [78] and inhibit the sidelobe level [79], and can also improve the ultrasonic emission intensity and increase the effective measurable distance.

### 2.3. Ultrasonic Propagation Characteristics 

When the ultrasonic wave propagates in the medium, its sound pressure intensity gradually decreases, known as attenuating. Attenuation is a result of several factors from interaction with medium and interfaces, including scattering, absorption, reflection and diffraction. In the ultrasonic ranging system where the transducer operates in pulse-echo mode, the transducer emits the ultrasonic wave and is reflected by the target obstacle, the ultrasonic overall loss formula can be expressed as shown in Equation (4), including the attenuation contribution from the propagation path [76]

(4) $$G=\frac{P_{r}}{P_{t}}=G_{ac}\frac{a}{4r}10^{-2\alpha r}$$

where *G* is ultrasonic attenuation coefficient, $P_{r}$ is the pressure of the ultrasonic wave received by the transducer, $P_{t}$ is the pressure of the ultrasonic wave emitted by the transducer, $G_{ac}$ is the acoustic gain, depends on the size of the target, *a* is effective membrane radius, *r* is the range to the target, α is attenuation coefficient which increases with the frequency of sound waves, and also depends on humidity, temperature and ambient pressure. The ultrasound attenuation in the air can be as high as 7 dB/m at 215 kHz at room temperature and 60% relative humidity in the air [63]. In general, the ultrasonic ranging system tends to use transducers that operate at frequency range < 300 kHz, and typically at frequencies around 40 kHz, in order to obtain a balance between the measurement distance of interest, the measurement resolution and the signal quality, e.g., SNR. 

## 3. Ultrasonic Ranging System and Its Evaluation Parameters

The ultrasonic transducer is the key component of the ultrasonic ranging system. The system measures the ultrasonic wave transmitted and received by the transducer and converts it into distance measurement to complete the function of the ranging system.

### 3.1. Principle of Ultrasonic Ranging

The ultrasonic transducer transmits ultrasonic waves and receives echo reflected from the object by either the same transducer, pulse-echo mode or by another transducer as pitch-catch mode. When using the pulse-echo mode, the distance *s* between the ultrasonic transducer and the object is to be calculated by measuring the time *t* between the transmitted signal and received signal as shown in Equation (5)

(5) s=ct/2

where *c* is the sound velocity in the medium.

**Figure 4:**
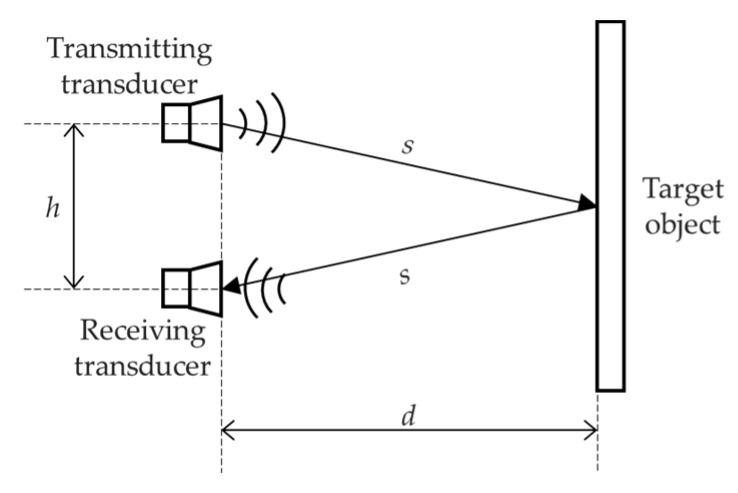
Schematic diagram of Ultrasonic Ranging with a pair of transducers.

Figure 4 shows a pair of the transducers are configured in pitch-catch configuration, the distance between the plane where ultrasonic transducers are located and the target object *d* is

(6) $$d=\sqrt{\left(s^{2}-{\left(h/2\right)}^{2}\right)}$$

In practice, d is much greater than the distance between the two transducers h and therefore h can be treated as negligible. That means d=s and Equation (6) can also apply in this configuration. 

### 3.2. Composition of Ultrasonic Ranging System

**Figure 5:**
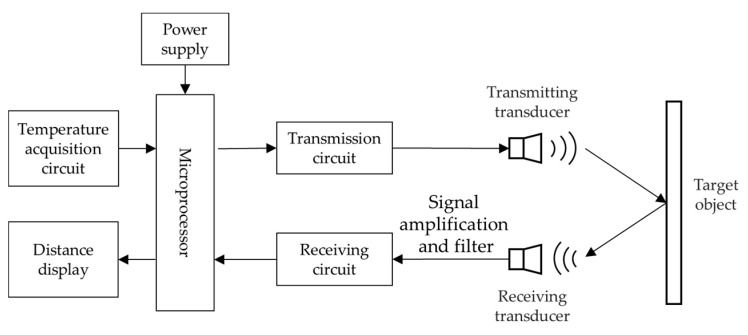
An overall diagram of ultrasonic ranging system.

A typical ultrasonic ranging system would be as shown in Figure 5 and composed of transducers for transmitting and receiving, transmitting circuits, receiving circuits, a microprocessor, a temperature compensation module and a display module [80,81,82,83]. 

Transducers include the transmitting transducer and the receiving transducer. The transmitting circuit amplifies the programmed pulses to high voltage pulses that can drive the transducer, while the preamplifier of the receiving circuit amplifies the received signals because they are generally weak [84]. Then, the signal passes through the bandpass filter to remove interfering noise signals and improve the SNR of received signals. To avoid the high requirements of the hardware, the bandpass sampling theory can be applied to digitalize the echo signal [85].

Microprocessor generally adopts a micro controller unit (MCU) which is high-performance and low-power [86,87,88]. The microprocessor controls the programming of the transmitting signal. At the same time, it also processes the received signal to obtain the information of interests and transmits the measurement results to the display module. Meanwhile, the power supply module is the basis of the normal work of the ranging system. 

**Figure 6:**
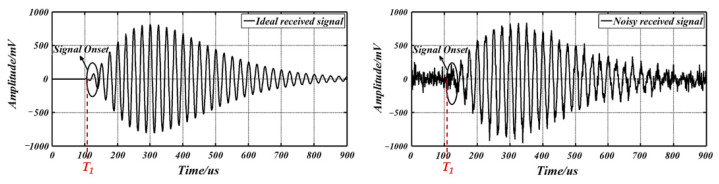
Ideal and noisy ultrasonic echo signals [90].

As for the processing of received signals by the microprocessor, the key is to obtain the starting time $T_{1}$ of echo signals, the ideal and noisy ultrasonic echo signals are shown in Figure 6. In addition, the amplitude of the received signal varies with the measured distance [89]. Different methods can be used to obtain $T_{1}$, which will be elaborated in detail in Section 4.

The temperature compensation module compensates for the influence of temperature on the propagation velocity of ultrasonic waves [80]. For every 1 °C increase in temperature, the speed of sound increases by about 0.607 m/s [91]. The circuit is equipped with a temperature sensor and connected with the microprocessor to realize the acquisition of real-time ultrasonic velocity.

Display module: For the measurement data obtained by the microprocessor, they can be directly connected to a liquid crystal display (LCD) for real-time display [92] or transmitted to other microcontrollers through wireless communication to avoid the inconvenience of reading the measurement data directly [93].

**Figure 7:**
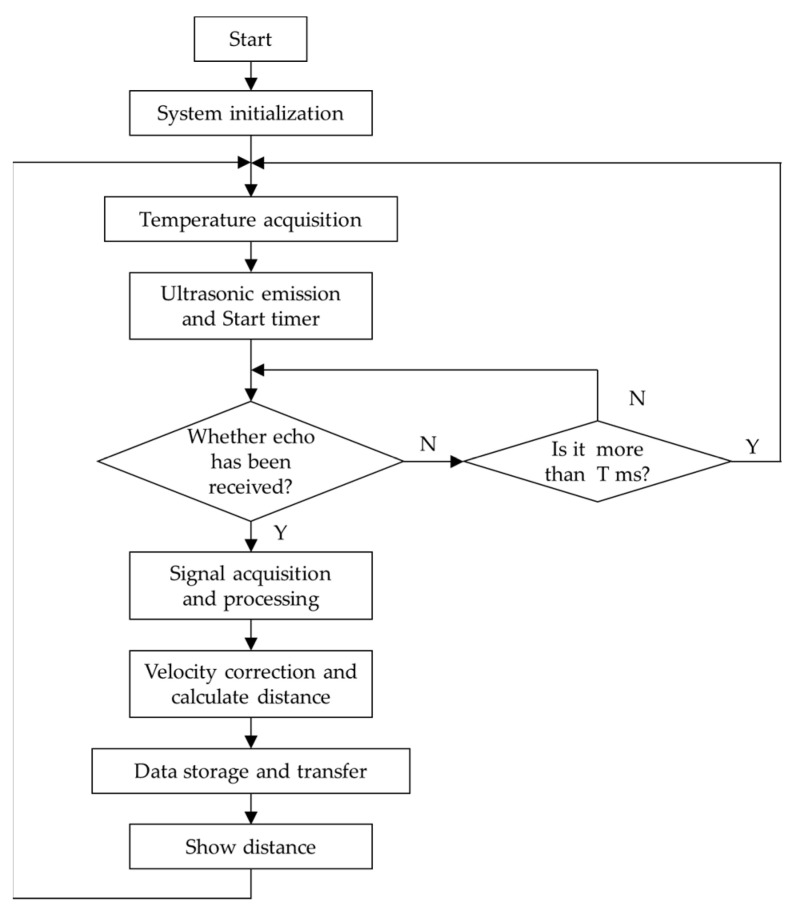
A program flow of an ultrasonic ranging system.

Several research groups had developed their own ultrasonic ranging system [80,82,94,95,96], and we extract and summarize the program flow of the ultrasonic ranging system and show in Figure 7. The value of T is set in direct proportion to the furthest measurement distance, that is, the single measurement period is inversely proportional to the value of T.

### 3.3. Evaluation Parameters of Ranging System 

#### 3.3.1. Measurement Range of Distances and Angles 

**Figure 8:**
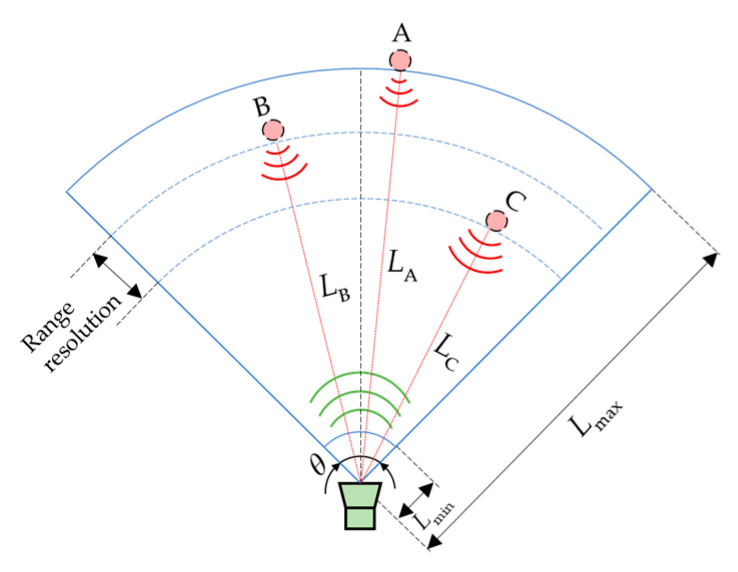
Schematic diagram of measuring range.

The sector in a two-dimensional space or the conical shape in a three-dimensional space can be represented as the effective range of ultrasonic ranging as shown in Figure 8 [97]. In the figure, θ is the beam spread angle which relates to the directivity of the transducer; $L_{\min}$ and $L_{\max}$ are the nearest and the farthest distance that can be measured, respectively. $L_{\min}$ is determined by the dead zone of the transducer, which is related to the near field of the ultrasound propagation. The dead zone is also related to the length of the excitation pulses [98]. Although the longer the excitation signal is, the more energy delivered and therefore the better signal quality at reception, its longer ringing down time will lead to a larger blind area. $L_{\max}$ is determined by the propagation attenuation characteristics of the ultrasonic wave described in Section 2.2.4. The measurement range of an ultrasonic ranging sensor is 2 cm–5 m in general to ensure adequate echo signal at reception [99].

In order to reduce the dead zone effect on the measurable range, the pitch-catch mode can be adopted with separate transmitters and receivers [100]. In this configuration, the pulse’s ringing down will not mask the echo received, therefore, reducing the dead zone $L_{\min}$. Another approach is to optimize the transmit pulse and receiving filter by customizing the transmit pulses of chirp 80–65 kHz with Cauer high-pass filter to reduce the receive pulse duration and combining with partial ringing removal by using ringing look-up-tables to reduce the minimum detectable range down to 3 cm [101]. In addition, to increase $L_{\max}$ and angle range, a transducer array can be adopted [102].

#### 3.3.2. Measurement Accuracy of Ultrasonic Ranging System 

The measurement accuracy of distance *s* depends on the accuracy of both transmit time *t* and sound velocity *c*. Among them, the acquisition of transmit time is more critical, as detailed below.

First, when the direct transit time method is used, the accuracy of transmit time is determined by the accuracy of identifying the received time of ultrasonic waves. 

Secondly, phases of the propagated ultrasound waves can also be measured to extract timing information for distance calculation. When the phase method and the frequency modulated continuous waves (FMCWs) method are adopted, the transit time is essentially obtained indirectly. The accuracy of distance is also related to the range resolution or axial resolution [103]. It is the minimal range difference (the axial distance between the measured points B and C in Figure 8) needed to distinguish the movement of a target along one bearing. Therefore, the range resolution is the highest accuracy that can be achieved by ultrasonic ranging systems.

When the phase method is adopted, the distance corresponds to the phase value, and its range resolution can be expressed as

(7) $$\sigma_{1}=\frac{c\cdot \theta_{0}}{f\cdot 360{}^{\circ}}$$

where $\sigma_{1}$ is the distance resolution of the phase method, $\theta_{0}$ is the phase resolution, *c* is the sound velocity and f is the transducer frequency.

**Figure 9:**
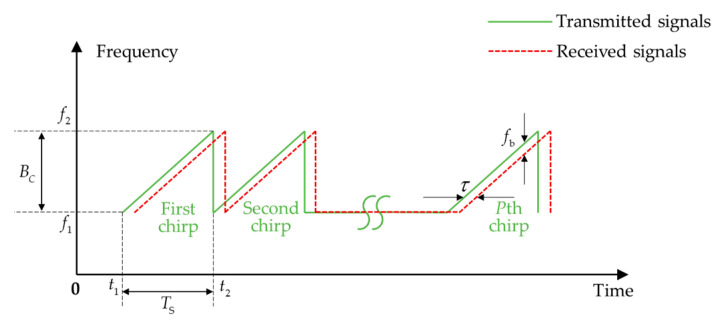
The transmitted and received chirp signals ($T_{s}$ is the scanning period of one chirp which is the difference between $t_{1}$ and $t_{2}$, $B_{c}$ is the scanning bandwidth which is the difference between $f_{1}$ and $f_{2}$, τ is time delay of received signals compared to transmitted signals and $f_{b}$ is the difference frequency between the transmitted signals and received signals).

FMCWs, also called chirps, are commonly used to enlarge the range of interest and improve measurement resolution, first in the radar [104], and later in ultrasonic nondestructive testing applications [105,106], ultrasonic medical imaging [107] and ultrasonic ranging [108]. The transmitted and received signals can be expressed as shown in Figure 9.

The range resolution of the FMCWs method is

(8) $$\sigma_{2}=\frac{c}{2B_{c}}$$

where $\sigma_{2}$ is the distance resolution of the FMCWs method, c is the sound velocity and $B_{c}$ is the scanning bandwidth.

The range resolution of the FMCWs method depends on the scanning bandwidth. Therefore, high bandwidth transducers are required for high range resolution. 

It is important to note that range resolution is the measurement accuracy in an ideal world. The accuracy in practice will be affected by many other aspects such as the hardware and the echo signal processing method. 

#### 3.3.3. Measurement Rate

The measurement rate is the reciprocal of the time required for a single measurement. When a single transducer is used in the ranging system to perform in pulse-echo mode, the transducer would usually be set up in a way that it will not fire the next pulse until a reflected echo is received. 

The maximum measurement rate of the pulse-echo method is as follows:

(9) $$\mathrm{Maximum}\;\mathrm{measurement}\;\mathrm{rate}=\frac{1}{t_{tof}}=\frac{c}{2\cdot d}$$

**Figure 10:**
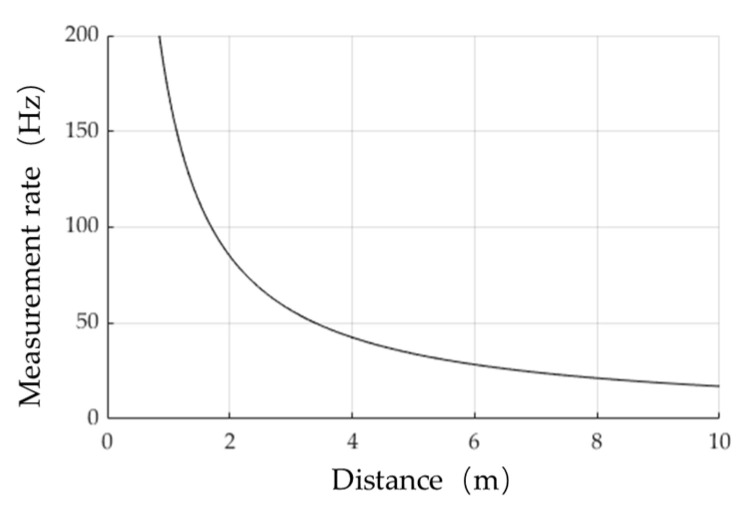
Diagram of the measurement rate and distance.

The measurement rate decreases inversely with the distance, and the relationship between measurement rate and measurement distance is shown in Figure 10. For a target of 5 m, the measurement rate is only 34 Hz, which cannot meet the requirements of measurement speed on all occasions such as the dynamic measurement of the blade tip distance between the upper blades and the lower blades. 

In summary, the performance of an ultrasonic ranging system depends on the measurement range, measurement accuracy and measurement rate, and should be designed according to the actual measurement requirements.

## 4. Ultrasonic Ranging Methods and Signal Processing 

Ultrasonic ranging methods are divided into the time of flight (ToF) method, two frequency continuous wave (TFCW) method and multi-frequency continuous waves (MFCW) method, binary frequency shift keying (BFSK) method, amplitude modulation method and signal coding method according to the measured parameters and the type of transmitted wave. The following subsections describe these methods in detail.

### 4.1. Time of Flight (ToF) Method

ToF method is the most common method. The transmitter sends simple single-frequency sequence pulses, and the receiver processes the echo signal to get the time of flight of ultrasonic waves, and then the distance value can be obtained by combining it with the sound velocity. The key for the ToF method is to obtain the time information accurately and that can be achieved through the amplitude threshold, envelope fitting and correlation method. 

#### 4.1.1. Amplitude Threshold Method (ATM) 

ATM is the most commonly used due to its simplicity and easy implementation, fast calculation speed and low hardware price [26,109].

**Figure 11:**
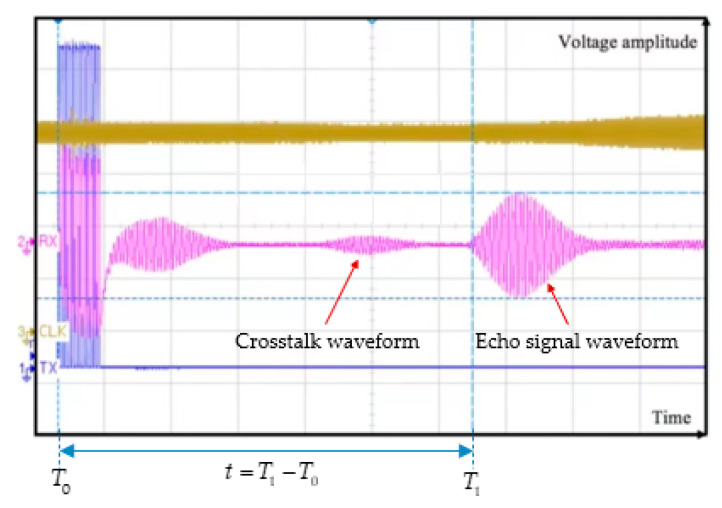
Driving and received signals with crosstalk (the brown line indicates the waveform of the system clock, the blue line indicates the twelve electrical pulse excitation signals and the purple line indicates the waveform of the receiver) [110].

Examples of driving and received signals are shown in Figure 11. The initial time of the driving signal is denoted as $T_{0}$, and the amplitude detection of the received signal is performed according to the amplitude threshold τ. In general, the amplitude threshold is set at 3 to 5 times the noise level due to the presence of noise [26]. When the amplitude reaches the threshold τ, the time is denoted as $T_{1}$, and the flight time is $t=T_{1}-T_{0}$. In practice, the receiver may have an electrical signal fluctuation before the ultrasonic echo arrives due to the crosstalk, as shown in Figure 11, which can be filtered out by hardware isolation or filtering algorithms. 

The performance achieved by the ranging system using the ATM is summarized in Table 2.

Compared with conventional bulk transducers, the maximum range of the PMUT is limited, generally about 1 m. As can be seen from Table 2, the shorter the measurement range is, the higher the accuracy can be achieved due to the better SNR of the echo signal.

In practice $T_{1}$ detected from the received signal presents an error and does not represent the exact arrival time of the echo signal. This is because of the presence of noise and the setting of the amplitude threshold. This error cannot be compensated as a systematic error because it is different when the propagation distance is different. In addition, this method is susceptible to noise, and the system will process it as an echo signal when the noise of a high level occurs occasionally. To solve this problem, ref. [113] proposed the double threshold method, also known as the sliding window method. A window of width N shifts along the echo signal one sample at a time. At each window position, calculate the number of samples exceeding the set threshold τ. If this number exceeds the second threshold m, then estimate ToF. The advantage of this method is its robustness to noise peaks because the detection of the target is based on m samples rather than a single sample with a single threshold. A measurement accuracy of 0.69 mm in the range of 100–600 mm is reported in [113].

#### 4.1.2. Envelope Fitting Method

In order to solve the problem of the ATM method in detecting the initial time of the echo signal, the curve fitting method can be used to fit the envelope of the echo signal, to find the starting point of the echo signal and generate the unbiased estimator of ToF.

**Figure 12:**
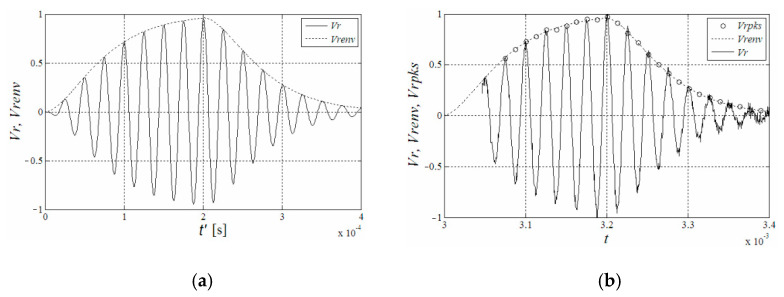
(**a**) An ideal model of the received signal, (**b**) An example of the fitting curve [114].

The ideal model of the received signal analyzed in [114] is shown in Figure 12a, where $V_{r}$ is the received signal and $V_{renv}$ is the envelope of the received signal. 

The envelope curve is simply fitted by the peak amplitude and time of each cycle, as shown in Figure 12b, or adopt the parabolic model [26]. Take the time corresponding to the point where the amplitude of the envelope curve is 0 as the initial time of the received signal.

With the above method, [114] reported the measurement accuracy can reach 0.7 mm within the range of 3000 mm distance and 0.3 mm within the range of 1000 mm. It can reach higher accuracy within the same range compared with ATM method in Table 2. In practice, the received signal envelope has deviation due to the influence of device performance, noise and other factors. Therefore, the envelope fitting method is more limited in actuality.

#### 4.1.3. Correlation Method 

The correlation method is considered the optimal TOF estimation technique in general [115]. It performs a cross-correlation calculation on the received echo signal and the transmitted signal, and then determine the flight time according to the maximum value of the cross-correlation signal. For a given sequence of transmitted and received signals $y_{p}(kT_{S})$ and $y_{E}(kT_{S})$, where $T_{S}$ is the sampling period, then the cross-correlation signal $X_{C}$ is

(10) $$X_{C}=\sum_{-\infty }^{+\infty }y_{p}(kT_{S})\cdot y_{E}(kT_{S}+nT_{S})$$

The ToF can be determined according to the peak position of $X_{C}$, and the peak lag is proportional to ToF, thus obtaining the measured distance *R* [116,117].

(11) $$R=(\tau_{\max}\cdot T_{s}-TOE)\cdot c-R_{cal}$$

where $\tau_{\max}$ is the lag of the maximum peak which is proportional to the time of arrival (TOA), *TOE* is the time of emission of the ultrasonic signal, and $R_{cal}$ is a calibration constant including all the fixed delays of the system which is independent of the range. Using the correlation method, the accuracy can reach 3.9 mm in the range of 30–450 mm with a PMUT of 214 kHz in [40,118] and 1.2 mm within the range of 2300 mm with the chirp of 15 kHz to 40 kHz [119]. In order to further improve the accuracy, the spline interpolation method can be adopted. However, the processing time will be increased. The accuracy can reach 0.25 mm in the range of 200–1000 mm, and the operation time needs 0.3 s in [120].

In addition, the flight time can be obtained through a combination of methods in order to improve the accuracy over a longer distance. There are studies that apply cross-correlation to correct the distance error for one wavelength scale and then use a phase-shift technique for subwavelength range refinement [121]. Reference [122] reported a 1 mm ranging resolution for the distance up to 3000 mm and [90] has achieved 0.5 mm accuracy for the distance up to 5000 mm.

In summary, ATM is simple and fast due to fewer processing requirements when compared to the envelope fitting and correlation algorithm, and therefore can be applied to the measurement of moving targets. The envelope fitting method has a processing speed of 6 ms to 8 ms in [114] and is limited by the envelope model. While the processing speed of correlation method is slower than ATM, the processing time can reach 0.7 ms of correlation method while 0.07 ms of ATM with the same processing device [123]. What should be kept in mind is that the measurement accuracy is not only related to the processing method but also related to the performance of the devices and measurement environment. The method of obtaining ToF should be selected according to the specific measurement requirements in measurement accuracy, processing speed and the speed of the measurement target. 

### 4.2. Two Frequency Continuous Wave (TFCW) and Multi-Frequency Continuous Waves (MFCW)

TFCW and MFCW methods obtain the time delay through phase difference measurement [124] and provide higher accuracy in measurement at the expense of measurement range when compared to the conventional ToF method. 

#### 4.2.1. Two Frequency Continuous Wave (TFCW)

To apply TFCW methods, the transmitter sends two excitation signals of the continuous wave with two frequencies of $f_{1}$ and $f_{2}$ ($f_{1}<f_{2}$) respectively, and the phase shifts of the two signals can be measured respectively when the waves reach the receiver. Then the distance between transmitter and receiver can be expressed as

(12) $$d=\left(n_{1}+\frac{\theta_{1}}{2\pi }\right)\lambda_{1}$$

(13) $$d=\left(n_{1}+\frac{\theta_{1}}{2\pi }\right)\lambda_{2}$$

where $\lambda_{1}$ and $\lambda_{2}$ are wavelengths of transmitted signals at frequencies $f_{1}$ and $f_{2}$ respectively, $\theta_{1}$ and $\theta_{2}$ are the phase shifts of two received signals compared to transmitted signals respectively. Therefore, the phase shift can be expressed as

(14) $$\theta_{2}-\theta_{1}=∆\theta -2\pi \left(n_{2}-n_{1}\right)$$

where ∆θ is

(15) $$∆\theta =2\pi d\left(\frac{1}{\lambda_{2}}-\frac{1}{\lambda_{1}}\right)$$

The integers *n* have only two possible values: $n_{2}=n_{1}$ and $n_{2}=n_{1}+1$. The difference of phase shifts can be defined by the following Algorithm 1:
**Algorithm 1**1: If $n_{2}=n_{1}$, $\theta_{2}-\theta_{1}=\Delta \theta$2: If $n_{2}=n_{1}+1$, $\theta_{2}-\theta_{1}=\Delta \theta -2\pi$

Therefore, the measured distance *d* can be expressed as

(16) $$d=\frac{\Delta \theta }{2\pi }\cdot \frac{c}{\Delta f}$$

where $\Delta f=f_{2}-f_{1}$. Therefore, in a certain sound propagation medium, the measurement range depends on the phase shift difference and frequency difference. The range can be increased to the wavelength corresponding to Δf, and the accuracy is determined by the phase resolution. The highest resolution of the phase depends on the clock frequency of the hardware system; for example, when using a 40 MHz clock for phase counting of a 40 kHz signal, a maximum resolution of 0.1% is obtained [125]. In the actual measurement, the distance resolution can reach 1% wavelength [100,126].

References [127,128,129] all adopted the TFCW method, and the performance achieved by the measurement system is summarized in Table 3.

As can be seen from Table 3, TFCW is suitable for measuring a distance within a short range. In order to expand the range with two different frequencies waves, the phase detection method can also be combined with other methods such as the amplitude of the waveform which can achieve the resolution of 1.5% wavelength over the distance of 550–1450 mm [130,131]. 

#### 4.2.2. Multi-Frequency Continuous Waves (MFCW)

According to the ranging principle of TFCW, its minimum range resolution is c/(Δf·360°) (m/°), which means that a larger frequency difference can obtain a higher resolution but results in the decrease in the measuring range. Therefore, the TFCW method cannot meet the requirements of minimum resolution and maximum range at the same time. While the MFCW method derived from the TFCW method which uses three frequencies ultrasonic waves for range measurement can meet the requirements simultaneously. 

**Figure 13:**
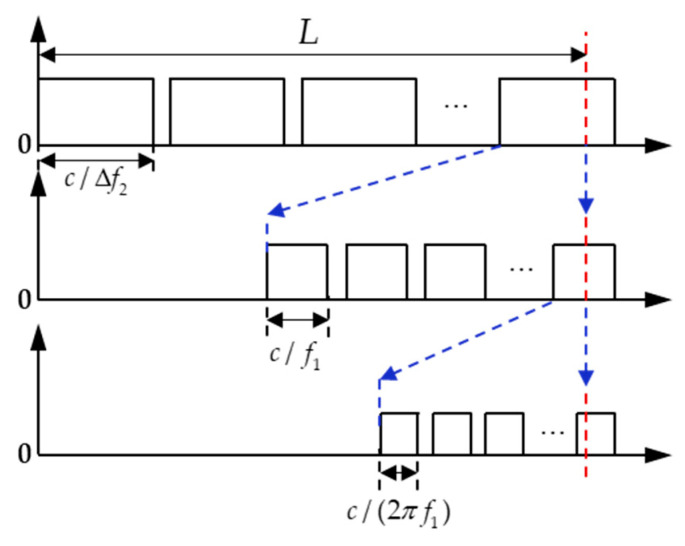
The working mechanism of the MFCW algorithm where *L* represented the overall distance to be measured between transmitter and receiver.

The working principle of MFCW in [132] is shown in Figure 13. Suppose that the frequencies of the transmitted continuous waves of different frequencies are $f_{1}$, $f_{2}$ and $f_{3}(f_{1}>f_{2}>f_{3})$ respectively, the phase shifts of the received signal and the transmitted signal are $\varphi_{1}$, $\varphi_{2}$ and $\varphi_{3}$ respectively. $\Delta \varphi_{1}$ is the phase difference between phase $\varphi_{1}$ and $\varphi_{2}$, and the corresponding frequency difference is $\Delta f_{1}(\Delta f_{1}=f_{1}-f_{2})$. Similarly, $\Delta \varphi_{2}$ is the phase difference between phase $\varphi_{1}$ and $\varphi_{3}$, and the corresponding frequency difference is $\Delta f_{2}(\Delta f_{2}=f_{1}-f_{3})$.

The calculation formula of the distance is [23]

(17) $$L=\mathrm{Int}\left[\frac{△\varphi_{1}}{2\pi }\cdot \frac{△f_{2}}{△f_{1}}\right]\frac{c}{△f_{2}}+\mathrm{Int}\left[\frac{△\varphi_{2}}{2\pi }\cdot \frac{f_{1}}{△f_{2}}\right]\frac{c}{f_{1}}+\frac{\varphi_{1}}{2\pi }\cdot \frac{c}{f_{1}}$$

The first step, yielding the largest resolution scale is determined by $c/\Delta f_{2}$(m/°). In the second step, yielding finer resolution is determined by $c/f_{1}$(m/°). In the final step, the highest level of resolution is determined by $c/(360{}^{\circ}\cdot f_{1})$(m/°). Taking FMCW of frequencies $f_{1}=40.0\;\mathrm{kHz}$, $f_{2}=39.9\mathrm{kHz}$, $f_{3}=38.0\mathrm{kHz}$ as an example, due to most commercial ultrasonic transducers having a narrow bandwidth of 40 ± 2 kHz [125], its highest resolution is 0.0243 mm/degree. 

References [23,132] use the MFCW method, and the performances of the two ranging systems are summarized in Table 4.

In conclusion, the MFCW method can achieve high accuracy within a larger range (1500 mm in Table 4) than the TFCW method (70~150 mm in Table 3). The measurement accuracy of both the TFCW and the MFCW depends on the phase measurement accuracy and the maximum range depends on the frequency difference. However, the measurement period becomes longer and the measurement rate is low due to the transmitter needing to transmit two or three different frequency signals successively.

### 4.3. Signal Modulation Method 

#### 4.3.1. Binary Frequency Shift Keying (BFSK) 

Binary frequency shift keying is similar to the conventional frequency modulation method except for its center or carrier frequency is shifted by the binary input signal which varies between logic 0 and logic 1.

BFSK signal can be expressed as [133]

(18) $$u(t_{i})=A\sum_{j=1}^{M}\Pi_{j}(t_{i})\sin[2\pi f_{b_{j}}(t_{i}-d_{j-1})]$$

where *A* is the amplitude of the transmitted signal, $b_{j}$ is the *j*th element of the binary 0,1 code sequence. When $b_{j}=0$, $f_{b_{j}}=f_{0}$, and when $b_{j}=1$, $f_{b_{j}}=f_{1}$. $f_{0}$ and $f_{1}$ are two different frequencies and the function $\Pi_{j}(t_{i})$ is defined as follows

(19) $$\Pi_{j}(t_{i})=\{\begin{array}{ll} 1 & for\;d_{j-1}\leq t_{i}\leq d_{j}\\ 0 & elsewhere \end{array}$$

and

(20) $$d_{j}=v\sum_{k=1}^{j}\frac{1}{f_{b_{K}}}\;\;\;\;\;\;\;\;j=1,2,3\ldots ,M$$

$d_{0}=0$. *v* represents the number of wavelengths corresponding to each bit of code value, which determines the duration of the total pulse $d_{M}$.

**Figure 14:**
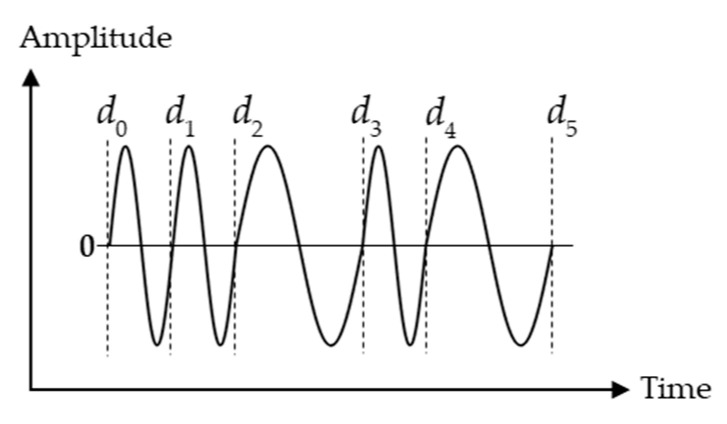
Transmitted BFSK signals with a binary code of 11010 ($d_{0}=d_{1}=d_{3}=0$ and the corresponding frequency is $f_{0}$, $d_{2}=d_{4}=1$ and the corresponding frequency is $f_{1}$).

An example of a transmitted BFSK signal is shown in Figure 14.

The transmitted signal can then be received and processed to obtain the ToF information and calculate the distance, using methods of the phase measurement method, correlation algorithm or the combination of different methods. 

**Figure 15:**
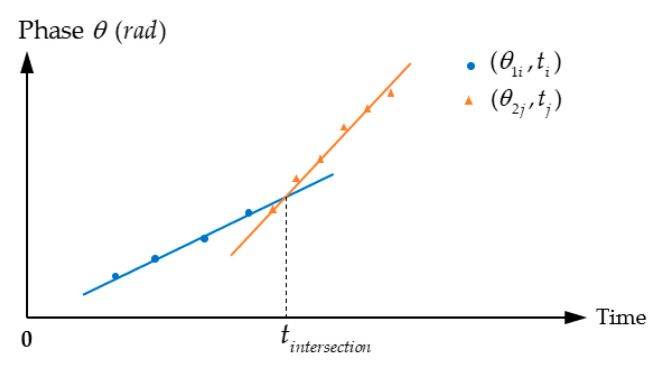
A function diagram of received BFSK signal phase verse time if using phase measurement method.

First, the phase measurement method can be adopted. The method in [24] is represented as shown in Figure 15. For example, when the transmitted BFSK signal is

(21) $$u(t)=\{\begin{array}{c} A_{s}\sin(2\pi f_{1}t)\;\;\;\;-t_{p}<t<0\;\\ A_{s}\sin(2\pi f_{2}t)\;\;\;\;0\leq t<t_{p} \end{array}$$

where $A_{s}$ is the amplitude of the transmitted signals, $f_{1}$ and $f_{2}$ are frequencies of transmitted signals respectively, $2t_{p}$ is the total pulse duration, without considering any distortion, the received signal can be expressed as

(22) $$r(t)=\{\begin{array}{c} A_{r}\sin[2\pi f_{1}(t-\tau )+\psi_{1}]+n(t)\;\;\;\;\;\tau -t_{p}<t<\tau \;\\ A_{r}\sin[2\pi f_{2}(t-\tau )+\psi_{2}]+n(t)\;\;\;\;\;\tau \leq t<t_{p}+\tau \end{array}$$

where $A_{r}$ is the amplitude of the received signals, τ is the time delay which is ToF, $\psi_{1}$ and $\psi_{2}$ are the phase shifts respectively, n(t) is Gaussian noise. Extracted from Equation (22), the phase of the two signals can be expressed as

(23) $$\theta_{1i}=2\pi f_{1}t_{i}-2\pi f_{1}\tau +\psi_{1}+\omega (t_{i})\;\;\;\;\tau -t_{p}<t_{i}<\tau \;,i=1,\ldots ,m$$

(24) $$\theta_{2j}=2\pi f_{2}t_{j}-2\pi f_{2}\tau +\psi_{2}+\omega (t_{j})\;\;\;\;\tau \leq t<t_{p}+\tau \;,j=1,\ldots ,n$$

where ω(t) is phase noise. The phase change can be expressed as shown in Figure 15 through linear regression of $\theta_{1}$ and $\theta_{2}$, and the intersection of the two lines $t_{intersection}$ can be expressed as

(25) $$t_{intersection}=\frac{b_{1}-b_{2}}{a_{2}-a_{1}}=\tau +K$$

where $a_{1}=2\pi f_{1}$, $b_{1}=-2\pi f_{1}\tau +\psi_{1}$, $a_{2}=2\pi f_{2}$, $b_{2}=-2\pi f_{2}\tau +\psi_{2}$. Equation (25) can be simplified as $t_{intersection}=\tau +K$, where $K=\frac{\psi_{1}-\psi_{2}}{2\pi (f_{2}-f_{1})}$ is a constant, and $t_{intersection}$ we get from Figure 15, so the ToF τ can be obtained. 

Second, correlation calculation can be adopted as polarity correlation function due to both of those signals are always a logic one or a logic zero, and is defined by [133]

(26) $$C_{uv}(\tau_{l})=\frac{1}{N}\sum_{i=1}^{N}\mathrm{sgn}[u(t_{i})]\mathrm{sgn}[v(t_{i+l})]$$

with $\tau_{l}=lT,l=0,1,2,\ldots ,L$, where T is the sampling time interval. $u(t_{i})$ is the transmitted signal, the returning echoes $v(t_{i})$ digitized by a comparator circuit and converted to a binary representation

(27) $$v_{k}(t_{i})=\{\begin{array}{c} 1\;\;\;\;\;\;\;for\;v(t_{i})>k\\ -1\;\;\;\;\;for\;v(t_{i})\leq k \end{array}$$

where *k* is the threshold set by the comparison circuit. The correlation signals are analyzed for peak detection to obtain the ToF. 

**Figure 16:**
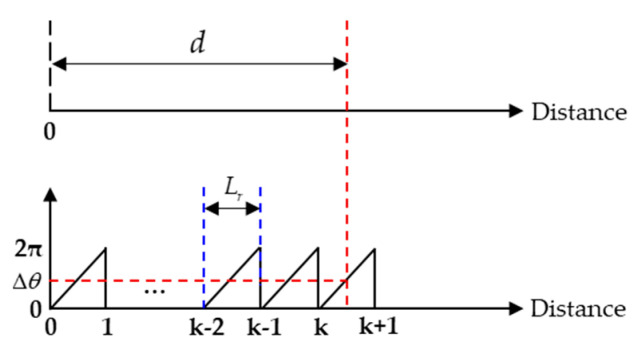
Relation between standard ToF method where d=(c·Δt)/2 and the combined method where d=1/2[(k−1)+(Δθ/2π)]·(c/Δf).

Third, the phase-shift detection method can be combined with the direct measurement of ToF to complete ranging [134]. The target distance is expressed as d=(c·Δt)/2, where Δt is ToF, *d* is divided into the regions as shown in Figure 16, $\left[(k-1)L_{r},kL_{r}]\;(k=1,2,3,\ldots \right)$, $L_{r}$ is the wavelength of frequency difference $\Delta_{f}$, then *d* can be expressed as d=1/2[(k−1)+(Δθ/2π)]·(c/Δf) since *k* is an integer. Therefore, the distance is

(28) $$d=\frac{1}{2}\left[\mathrm{Int}(\Delta t\cdot \Delta f)+\frac{\Delta \theta }{2\pi }\right]\cdot \frac{c}{\Delta f}$$

The performance achieved by different processing methods is summarized in Table 5. 

The BFSK method can improve the power of the received signal, which is beneficial to the signal processing process. Compared with the correlation algorithm, the phase shift method combined with ToF can obtain better measurement accuracy, which can reach 0.05 mm [134]. The BFSK method can be used to measure a longer range due to the outstanding energy characteristic. 

#### 4.3.2. Amplitude Modulation (AM) Method

AM method is to add different amplitude information to the ultrasonic transmitted signal to improve the measurement accuracy, measurement rate and other performances.

**Figure 17:**
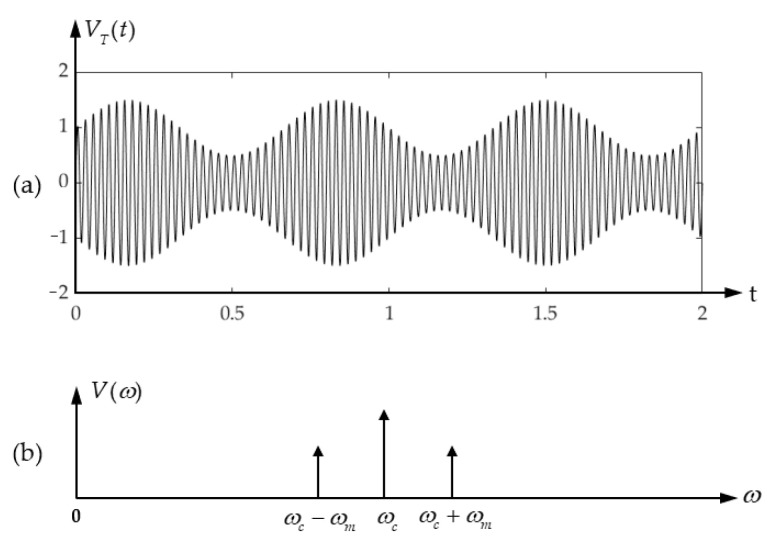
(**a**) Waveform and (**b**) spectral density of an amplitude-modulated signal.

The first application is the multifrequency amplitude modulation method (MFAM) [25] based on phase measurement. If the transmitter is excited by a continuous sine wave, the received echo signal can be expressed as

(29) $$V_{R}(t)=A\sin(\omega t+\varphi )$$

where *A* is the peak amplitude of the echo signal, ω is the resonant angular frequency, φ is phase shift which is proportional to the measures distance. Assume that both the carrier signal and the modulation signal are assumed to be sinusoidal signals with zero phase shift

(30) $$V_{T}(t)=A_{m}[1+m\sin(\omega_{m}t)]A_{c}\sin(\omega_{c})$$

where $A_{m}$ and $A_{c}$ are the peak amplitudes of the modulated signal and carrier signals, respectively; $\omega_{m}$ and $\omega_{c}$ are the angular frequencies of the modulated signal and carrier signals, respectively; *m* is the modulation index which defines the ratio of amplitude between modulating signal and carrier signal. The waveform and spectral density of the transmitted signal are shown in Figure 17 as an example. 

When the modulation index is less than 1, the envelope of the modulation waveform is proportional to the modulation signal, and the detected echo modulation signal is

(31) $$V_{R}(t)=A_{m}^{\prime }[1+m\sin(\omega_{m}t+\Delta \varphi_{m})]A_{c}^{\prime }\sin(\omega_{c}t+\Delta \varphi_{c})$$

where $\Delta \varphi_{m}$ and $\Delta \varphi_{c}$ are the phase shifts of the modulated signal and carrier signal, respectively. $\Delta \varphi_{m}$ and $\Delta \varphi_{c}$ are proportional to the measured distance *L*. When using phase-shift calculations of modulated signals, the distance *L* is

(32) $$L=\frac{\Delta \varphi_{m}\cdot c}{2\pi f_{m}}$$

When ${L/\lambda }_{m}<1$ the phase difference $\Delta \varphi_{m}$ provides a unique measurement distance *L* and the maximum measuring distance is $\lambda_{m}$ which is dependent on $f_{m}$. 

By adopting MFAM, the measurement range can reach the level of 10 m with the measurement accuracy in millimeters [137,138]. Since the distance information is obtained according to the envelope of the received signal, so the performance of the measurement system will not be affected by the loss of the initial part of the received signal due to it could be too weak to detect. The sinusoidal envelope of AM ultrasonic signals can be extracted real-time using the sliding discrete Fourier transform (SDFT) algorithm. In addition, for 25 Hz modulation, the present method shows a range of 13.72 m and an update rate of 3200/s for static objects. For moving objects, the update rate directly varies with the modulating frequency. The transient update rate for 25 Hz modulation is about 8/s in [138], so it is suitable for static and slow-moving targets measurement. 

The performance parameters that can be achieved by the ranging systems based on AM method are summarized in Table 6. 

**Figure 18:**
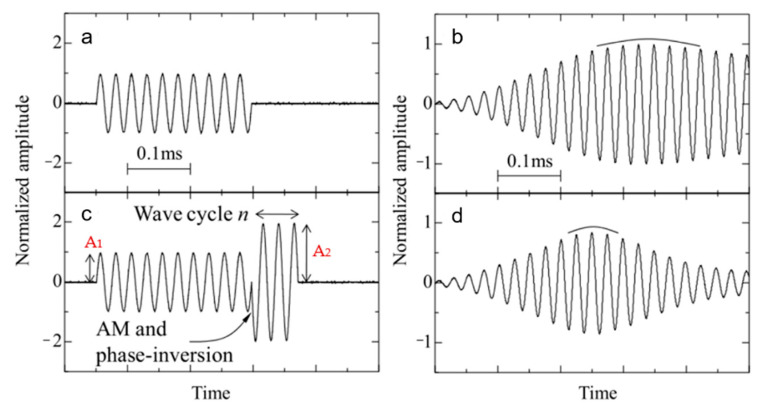
Ultrasonic waveform (**a**) Conventional driving signal and (**b**) its received waveform. (**c**) AMPI driving signal and (**d**) its received waveform [139].

The second application is the combination of amplitude modulation and phase modulation method (AM + PM) and obtains the ToF from the peak of the received signals. The peak of the received signal envelope is sharper through amplitude modulation and phase inverted (AMPI) of the transmitted wave, as shown in Figure 18. This is because several periods of signal with opposite phases are added after the conventional excitation wave to reduce the trailing part of the signal that is not expected to appear, and when the coefficient $k=A_{2}/A_{1}$ ($A_{1}$ and $A_{2}$ are the amplitudes of the AMPI driving signal) is larger, the effect is more obvious. Its sharp envelope improves the measurement accuracy of the peak time.

**Figure 19:**
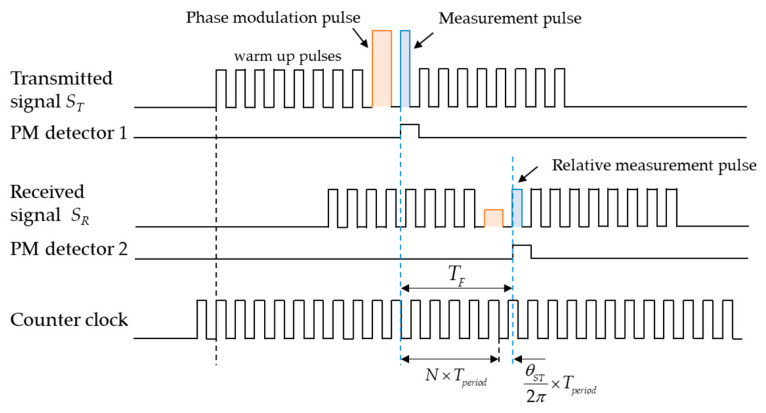
Examples of transmitted and received signals and ToF acquisition method.

Another specific modulation method of AM + PM is used in [140], to reduce the measurement error of ToF extraction caused by inertia delay and amplitude attenuation of the echo signal. The method is represented in this paper as shown in Figure 19. The transmitted signal *S_T_* consists of warm up waves and measurement waves. The first low-amplitude square waves are used to warm up transducers, which can eliminate the inertia delay caused by the piezoelectric effect of the ultrasonic transducer. The high pulse is the phase modulation and measurement pulse. The measurement pulse is 180° out of phase with the warm-up pulse. Therefore, the receiving signal can easily identify the position of the measured pulse. Here, the received signal *R_T_* is displayed in the form of the transformed square wave. Combined with the clock, the ToF $T_{F}$ can be obtained, which can be expressed as

(33) $$T_{F}=\left(N+\frac{\theta_{ST}}{2\pi }\right)T_{\mathrm{period}}$$

where *N* represents the integral part of the clock cycle, $\theta_{ST}$ is phase shift and $T_{\mathrm{period}}$ is the clock cycle.

By adopting AM + PM, the measurement accuracy of peak time of received signal can be improved due to the reduction of inertia delay. The performance of the ranging system in [139,140,141] is summarized in Table 6. It can be seen that the measurement accuracy can be very high in the short distance range, up to 0.02 mm, smaller than 0.3% wavelength, improving the ranging accuracy effectively. Therefore, MFAM is more suitable for the requirement of high update rate and larger range, while AM + PM is more suitable for the requirement of high accuracy and smaller range.

#### 4.3.3. Coded Signal Excitation Method

Signal coding is to add characteristics to transmitted signals, including frequency, phase, pulse position, pulse width, etc. It has recorded benefits to increase the SNR of the received signal, to reduce crosstalk between multiple sensors and to improve the measurement rate in long-distance measurement.

At present, the coding sequence used in the ranging system includes Chirp [142], Gold codes [143], Kasami codes [144], Golay codes and Loosey Synchronous codes [145], all of which improve the quality of the received echo signal. Encoding the transmitted signal and matching filtering at the receiving end can improve the performance of noise immunity due to the good auto-correlation property of the codes, thus improving the measurement resolution and measurement accuracy. 

**Figure 20:**
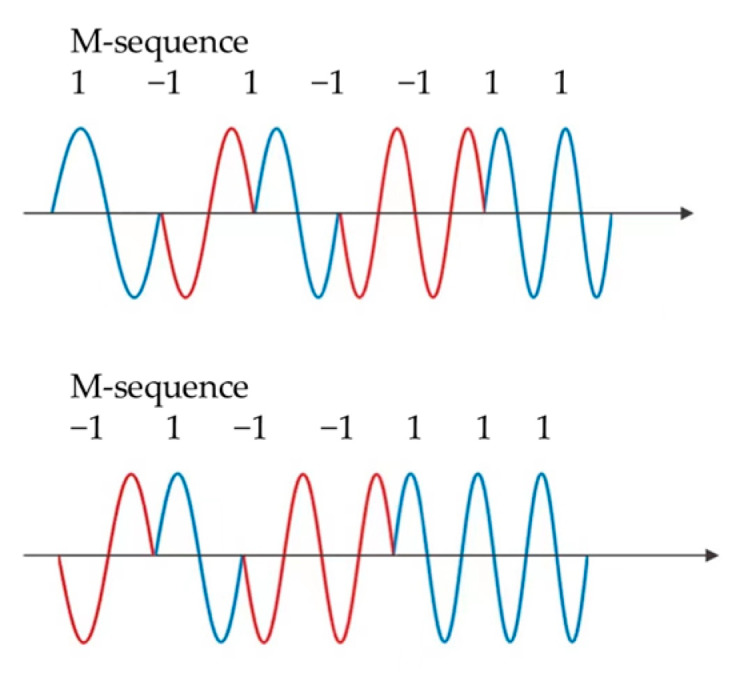
Two LFM signal examples coded by the same M-sequence but start from different sequence indexes.

In the case where there are multiple sensors in the ranging system, the crosstalk between sensors will cause confusion and damage to the system performance. Minimizing the crosstalk effects is a key problem in the field of ultrasonic measurement [146]. Therefore, it is necessary to add distinguishing characteristics to the signals sent by each sensor. The signals can be modulated by adopting e.g., Barker codes [147], Golay codes [148,149], M-Sequence [150], pulse position modulation [151,152] and pulse width modulation [153], even a combination of the multiple methods above. For example, M-sequence (which is one of the pseudorandom sequences generated from a linear feedback shift register (LFSR))combined with Chirp signals is shown in Figure 20. An M-sequence is a pseudorandom sequence of binary words composed of “1” and “−1”. An *n*th-order M-sequence is generated from an n-bit LFSR and its length is $2^{n}-1$ words. The binary words determine the phase and the Chirp signals determine the frequency of the transmitted signals. In the figure, code values of different positions in the same sequence are selected for encoding. The key to coding is to find codes with good autocorrelation and bad cross-correlation, so that each code has its own characteristics and the receiver can identify whether it is the signal sent by the corresponding transmitter. 

Third, the measurement rate of ultrasonic ranging is low in long-distance measurement due to the slow propagation speed of ultrasonic in the air, for instance, the measurement rate is only 17 Hz when the target distance is 10 m. Therefore, it is necessary to increase the measurement rate to make it suitable for high-speed measurement.

**Figure 21:**
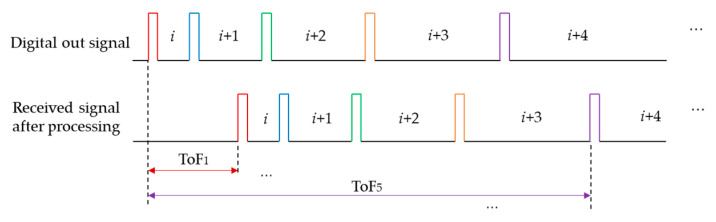
Pulse position modulation signals.

One approach to increase the measurement rate is through the application of pulse position modulation (PPM). The time interval between each pulse varies, linearly increasing in [154] shown in Figure 21. The pulse position characteristics will be transferred to the received signals and reflected by measuring ToF of each pulse to obtain distance. 

Reference [154] reported that they set the pulse time interval to increase from 3 ms to 11 ms, with a step of 1 ms. The average theoretical measurement rate can reach 143 Hz due to the average interval of 7 ms. In fact, according to the experiment results, the measurement rate of 4 m target can be increased by 2.7 times [154]. However, when the distance increases, there is a big difference between the actual measurement rate and the theoretical value, which is due to the influence of noise signal when the distance increases.

**Figure 22:**
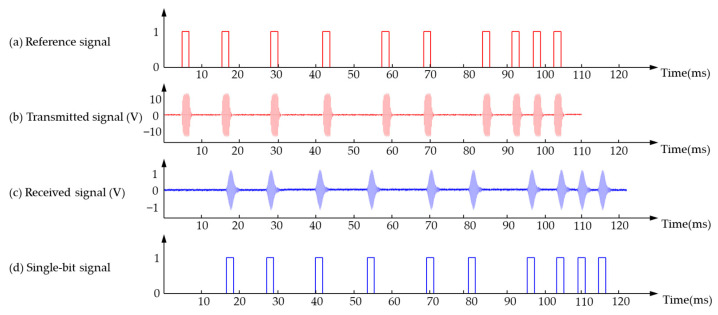
Signal processing procedure. (**a**) Reference signal; (**b**) Transmitted signal (V); (**c**) Received signal (V); (**d**) Single-bit signal.

Chaotic pulse position modulation (CPPM) [155] is another approach that is derived from PPM. The CPPM method was used to increase the measurement rate in [156]. The method is represented in this paper as shown in Figure 22. The chaotic information of the analogue voltage is transferred into the intervals between digital pulses. The received signals are converted into pulse signals as shown in Figure 22d and then cross-correlated with single-bitted transmitted reference signals as shown in Figure 22a to obtain the measured distance of each pulse. 

A measurement rate greater than 100 Hz can be obtained up to the distance range of 9 m, reported in [156], which effectively improves the measurement rate compared to the traditional pulse-echo method. The performance of this method is better than PPM. This also shows that this correlation method can extend the ranging distance to 5–10 m whilst still obtaining a better result of the measurement rate. 

In conclusion, ToF is the basic method, usually using ATM and correlation methods for data processing. TFCW and MFCW adopt several signals of similar frequency for ranging, and data processing is based on phase detection to improve the accuracy in a short-range. BFSK and AM methods modulate transmitted signals which can improve the quality of received signals to improve the measurement range or measurement accuracy. The signal coding method is to add identifiable features to different transmitted signals, which can solve the problem of multi-sensor crosstalk and improve the measurement rate in the case of long distances. 

## 5. Ranging Error Analysis and Compensation

The source of ranging error is mainly divided into two parts, one is the system error such as the acquisition error of ToF, the other is the external environmental error such as sound velocity variation due to temperature, humidity.

### 5.1. System Acquisition Error and Compensation

The acquisition of ultrasonic ToF is related to the quality of the echo signal and the processing method. Under the premise of the same processing method, the better the quality of the echo signal, the more accurate the acquisition of ToF. 

First, the error is caused by the noise of the ultrasonic echo signal. Signal filtering such as Kalman filtering [157] can be used to remove the irrelevant signals, and then improve the SNR of the echo signal. The coded signal excitation method in Section 4.3.3 can also improve the performance of noise immunity. In addition, increasing the transmitting signal intensity can improve the strength of the received signal, thus reducing the error of ToF acquisition caused by noise.

**Figure 23:**
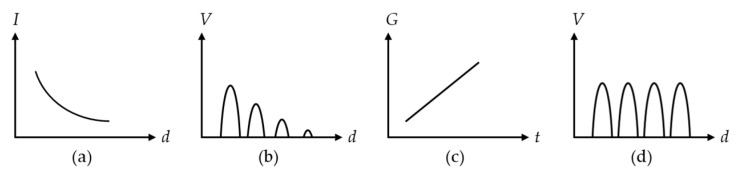
Gain compensation (**a**) Sound intensity varies with distance; (**b**) The relationship between received signal intensity and distance; (**c**) Compensation coefficient; (**d**) The relationship between received signal intensity and distance after compensation.

Second, the range error will increase with distance due to the significant attenuation of echo signal amplitude in the air. Therefore, a gain compensation module [80,158] can be added inside the receiving circuit to amplify the echo amplitude and compensate for the attenuation of ultrasonic energy. The compensation principle in [80] is shown in Figure 23, which makes the echo signal intensity of different distances consistent and reduces the acquisition error of ToF caused by subsequent signal processing due to different signal intensities.

**Figure 24:**
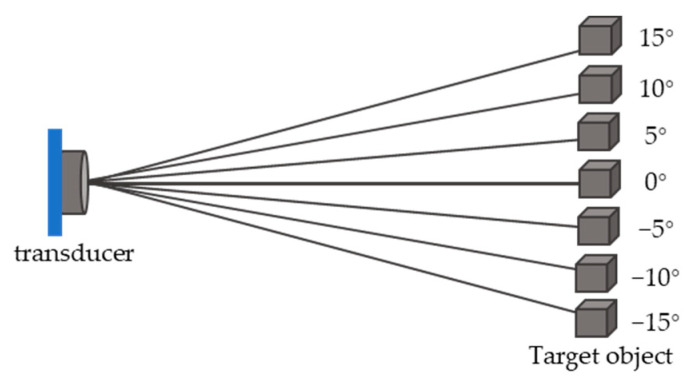
Angular range of target object detection.

Finally, the error is caused by the measurement angle within the detectable range of the beam spread angle θ. When the position of the target and the transducer present different angles which are shown in Figure 24, the ranging errors are different. The greater the angle is, the greater the ranging error will be [159]. The waveform of the ultrasonic wave away from the center line of the main beam, shown in Figure 3, will be distorted which makes the accurate echo time to be more difficult to identify. The detection angle should either be controlled, or the error can be compensated by a compensation algorithm which can be established through pre-measurement with sensors located at different angles. Reference [160] reported an improvement of the accuracy from 12.24 mm to 3.4 mm within 1 m at 60° angle position with angle compensation.

### 5.2. Environmental Error and Compensation

The sound velocity is easily affected by environmental factors, including temperature, humidity, pressure, etc. As a nonlinear function of temperature, pressure, humidity and CO_2_ concentration, sound velocity can be expressed as [161]

(34) $$c=f(t_{C},p,x_{w},x_{c})$$

where $t_{c}$ is Celsius temperature, *p* is the air pressure, $x_{w}$ the water vapor mole fraction, and $x_{c}$ is the carbon dioxide mole fraction. 

The temperature has the greatest influence on sound velocity, which can be expressed by the following formula [91]

(35) $$c=331.45\sqrt{1+\frac{t_{C}}{273.15}}$$

where 331.45 m/s is the sound velocity in dry air at 0 °C, $t_{C}$ is the temperature in degree Celsius. As temperature increases, the sound velocity increases. Equation (35) adopts Taylor series expansion and can be simplified as

(36) $$c=331.45+0.607t_{C}$$

For every 1 °C increase in temperature in air, the speed of sound increases by 0.607 m/s.

**Figure 25:**
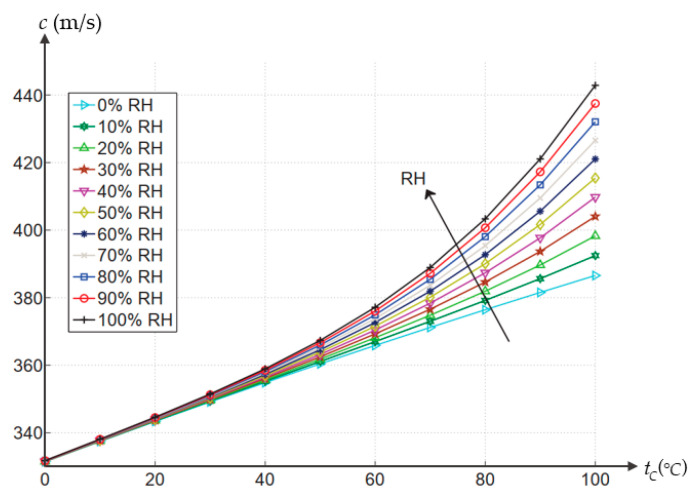
Variation of the speed of sound to temperature and relative humidity [162].

The influence of humidity on the sound velocity is inferior to that of temperature and can be expressed by the following formula [91]

(37) $$c^{2}=\gamma \frac{RT}{M}\left(1+\frac{2pB}{RT}\right)$$

where *c* is sound velocity, *T* is the temperature on an absolute scale, γ,p,R,M,B represent the specific heat ratio, pressure, the universal gas constant, molecular mass and second virial coefficient respectively. The speed of sound is faster in moist air than in dry air. The variation of the speed of sound with respect to temperature and relative humidity is shown in Figure 25.

The variations of air pressure and CO_2_ concentration have small influences on the speed of sound which can be negligible when compared to the effects caused by temperature and humidity. In the extreme humidity(100% RH) condition, there is a worst scenario for the pressure’s effect on sound speed as pressure’s influence increases with humidity, and where the maximum relative variation of sound speed caused by air pressure within [50,110] kPa is 0.67% at the temperature of 30 °C and CO_2_ concentration at normal level (Xc = 0.000383) [161]. The influence of CO_2_ concentration on sound speed is even smaller, about 0.06% relative variation of sound speed in 19 °C with a 0.2% mole fraction change in CO_2_ concentration [161,163].

In practice, the ranging system only compensates for errors caused by temperature and humidity. The temperature sensor module and humidity sensor module can be set inside the ranging system to measure the value of environmental parameters in real-time and compensate the sound velocity value accordingly. The sound velocity can also be measured in a real-time fashion alongside the main system, with a target set up at a known exact distance. The ToF information extracted from the target can be used to calculate the sound velocity under current conditions [164]. The advantage is that it can compensate for the sound velocity errors caused by a variety of environmental factors simultaneously. The disadvantage is that the space occupied by the ultrasonic ranging device will increase. 

In addition, multiple sensors can be set for measurement [165] or the time of flight ratio of different transducers can be used to obtain the positioning parameter [166]. The final measurement result can also be obtained through multiple measurements by a single sensor [167,168] to enhance the measurement reliability. For example, M-sequence can also be used to improve the ToF estimation accuracy as it measures the ToF of each pulse of the echo signal, and [168] reported that through averaging the 50 measures the error is reduced to one-tenth of one measure.

## 6. Conclusions and Outlook

The present status of ultrasonic ranging is reviewed, evaluated and discussed in this paper. 

First, the characteristics of ultrasonic transducers are classified, and their relationship to the ranging application is analyzed. To increase the energy transfer efficiency of the transducer, the working frequency of the transducer is selected as close as possible to the series resonant frequency, and the matching circuit is designed according to its impedance characteristics. The range of ultrasonic ranging depends on the directivity and propagation attenuation characteristics of the transducer. The higher the electromechanical coupling coefficient is, the lower the power consumption of the ranging system will be. 

Second, the principle of ultrasonic ranging, system composition and evaluation parameters are summarized. Ultrasonic ranging is generally measured by the pulse-echo method. The ranging system includes transducers, transmitting circuits, receiving circuits, a microprocessor, compensation modules, etc. The performance of the ranging system is evaluated according to the measurement range, measurement accuracy and measurement rate.

Third, the method of ultrasonic ranging and its signal processing are classified, and the performance of the measurement system is analyzed. The measurement results of the ToF method can be obtained by ATM, envelope fitting and correlation method. Among them, ATM is the simplest, with the fastest processing speed and a wider range of applications. The envelope fitting method has lower processing speed and higher theoretical measurement accuracy but is limited by the envelope model. The processing speed of the correlation method is average, but the overall performance is better. TFCW and MFCW methods use multi-frequency measurement signals based on phase detection to obtain results. Compared with the ToF method, the measurement range is smaller, and the measurement period is longer, but the measurement accuracy is higher. BFSK and AM methods modulate the transmitted signals which can improve the quality of received signals, thus improving the measurement range or measurement accuracy. The BFSK method controls the frequency of transmitted signals through binary coding and the accuracy of the method can reach 0.05 mm within 5 m. The AM method modulates the amplitude of the transmitted signals and the accuracy can reach 0.02 mm within 0.5 m. The signal coding method can suppress crosstalk between multiple sensors and improve the measurement rate of long-distance (10 m) measurement.

Finally, the ranging error and compensation methods are summarized. The ranging error includes the error of ToF estimation and the error of sound velocity. The error of ToF is compensated for by signal filtering, gain compensation of ultrasonic propagation attenuation and compensation algorithm of different measurement angles. The sound velocity error can be compensated for by setting an environmental parameter sensor or a known exact distance compensation module to improve the performance of the ranging system.

The application of ultrasonic ranging is increasingly developing due to its unique characteristics such as low hardware requirements, not being sensitive to ambient light and electromagnetic interference, and the low cost of transducers. With the continuous progress of ultrasonic ranging technology in transducers, measurement system configuration, measurement methods and signal processing, error compensation methods, we predict that the performance of ultrasonic ranging will present the following development trends:

*Adoption of MUTs*. MUTs will be more widely used in ultrasonic ranging systems with their advantages, such as small size, low power consumption, low cost, mass production and integration with other electronics. 

*Optimization of the transducer performance*. At present, the electromechanical coupling coefficient of PMUT is relatively low. The increase in the electromechanical coupling coefficient will reduce the power consumption of the system and improve the measurement range. In addition, the improvement of the transducer bandwidth will improve the working frequency range of the transducer and make its signal modulation more flexible, thus improving the overall performance of the ranging system.

*Improvement of the measurement range*. Performance improvements include the minimum detectable range and the maximum detectable range. At present, the blind area of ultrasonic ranging is large, which is at the level of centimeters. Optimization of devices and hardware circuits to reduce the blind area whilst extending the measurement range at the far end is the direction of future development. In addition, multi-sensor arrays and optimization of the transducer mentioned above will increase the maximum detectable distance and direction angle.

*Development of the processing algorithm*. The future development direction is multi-algorithm fusion processing of ultrasonic received signals so that the measurement accuracy and processing time can reach a balance with a higher comprehensive level.

*Applications in high dynamic and complex multi-target measurement*. At present, the number of detectable targets is limited and ultrasonic ranging is mostly used in the static or low dynamic measurement of simple targets, but in the future, high dynamic and complex multi-target measurement problems will be solved through better algorithms. 

*Improvement of ultrasonic measurement rate*. At present, there are few studies in this area. The method to improve the measurement rate is to encode the ultrasonic signal and realize the continuous transmission of the ultrasonic signal to overcome the limitation of the slow propagation speed of the ultrasonic signal in the long-distance measurement. On the other hand, the research of new coding technology will also make the comprehensive performance of the coded ultrasonic more superior, improve the measurement rate, to be more suitable for high-speed measurement requirements. 

In short, the performance of ultrasonic ranging technology will be further improved, and the application range will be wider in the further.

## Figures and Tables

**Table 1:** The characteristics of bulk piezoelectric transducers, CMUTs and PMUTs [45,51,52,53,54,56,57,58,59].

	Bulk Piezoelectric	CMUTs	PMUTs
Fabrication methods	Mechanical machining, e.g., dicing, lapping and casting	Wafer bonding and micromachining	Micromachining and wafer transfer diaphragm formation
Matching layer	Required	No matching layer	No matching layer
$k^{2}{}_{eff}$ * and bandwidth	Low	High	Low
CMOS compatible and flip-chip integration	No	Yes	Yes
DC bias requirement	No	Yes	No
General Size	Centimeter to millimeter level size	Millimeter level size of transducer arrays Hundred microns level size of a single CMUT	Millimeter level size of transducer arrays Hundred microns level size of a single PMUT

* $k^{2}{}_{eff}$: electromechanical coupling coefficient.

**Table 2:** Performance comparison of several ATM-based ranging systems.

Reference	Range	Accuracy	Transducer Type
[63]	1300 mm	1.3 mm	PMUT with 215 kHz
[110]	500 mm	0.63 mm	PMUT with 97 kHz and 96 kHz
[111]	1000 mm	4 mm	PMUT with 77.34 kHz
[109]	5000 mm	4 mm	Conventional bulk transducers with 35 kHz
[112]	100 mm	0.5 mm	Conventional bulk transducers with 40 kHz

**Table 3:** Performance comparison of several TFCW based ranging systems.

Reference	Range	Accuracy	Signal Frequency
[127]	30 mm~100 mm	1.5 mm	39.85 kHz and 40.6 kHz
[128]	50 mm~200 mm	0.1362 mm	40 kHz and 40.82 kHz
[129]	10 mm~110 mm	2.5 mm	94.21 kHz and 95.59 kHz

**Table 4:** Performance comparison of several MFCW based ranging systems.

	Range	Accuracy	Signal Frequency
[23]	1500 mm	0.05 mm	40.0 kHz, 39.9 kHz and 38.0 kHz
[132]	<100 mm	0.0711 mm	497.0 kHz, 496.8 kHz and 487 kHz
	100 mm~300 mm	1.8208 mm	492 kHz, 491.8 kHz and 490 kHz

**Table 5:** Performance comparison of several BFSK based ranging systems.

Reference	Range	Accuracy	Signal Processing Method
[24]	~5 m	1 mm	Phase measurement
[135]	0.5 m~4 m	/	Correlation algorithm
[136]	~7.2 m	6.2 cm	Correlation algorithm
[134]	~6 m	0.05 mm	ToF and phase shift

**Table 6:** Performance comparison of several AM based ranging systems.

Reference	Modulation Method	Range	Accuracy
[137]	MFAM	~1.5 m	2 mm
[138]	MFAM	~13.72 m	
		~3 m	1.343 mm
[139]	AM + PM	0.1 m~0.5 m	0.02 mm
[140]	AM + PM	0.05 m~0.5 m	0.2 mm
[141]	AM + PM	0.05 m~1 m	0.06 mm
		~2 m	0.15 mm
